# Plasticity in One Hemisphere, Control From Two: Adaptation in Descending Motor Pathways After Unilateral Corticospinal Injury in Neonatal Rats

**DOI:** 10.3389/fncir.2018.00028

**Published:** 2018-04-12

**Authors:** Tong-Chun Wen, Sophia Lall, Corey Pagnotta, James Markward, Disha Gupta, Shivakeshavan Ratnadurai-Giridharan, Jacqueline Bucci, Lucy Greenwald, Madelyn Klugman, N. Jeremy Hill, Jason B. Carmel

**Affiliations:** ^1^Motor Recovery Laboratory, Burke-Cornell Medical Research Institute, White Plains, NY, United States; ^2^Departments of Neurology and Pediatrics, Brain and Mind Research Institute, Weill Cornell Medicine, Cornell University, New York, NY, United States

**Keywords:** plasticity, corticospinal tract, rubrospinal tract, deficits, neonatal pyramidotomy, rats

## Abstract

After injury to the corticospinal tract (CST) in early development there is large-scale adaptation of descending motor pathways. Some studies suggest the uninjured hemisphere controls the impaired forelimb, while others suggest that the injured hemisphere does; these pathways have never been compared directly. We tested the contribution of each motor cortex to the recovery forelimb function after neonatal injury of the CST. We cut the left pyramid (pyramidotomy) of postnatal day 7 rats, which caused a measurable impairment of the right forelimb. We used pharmacological inactivation of each motor cortex to test its contribution to a skilled reach and supination task. Rats with neonatal pyramidotomy were further impaired by inactivation of motor cortex in both the injured and the uninjured hemispheres, while the forelimb of uninjured rats was impaired only from the contralateral motor cortex. Thus, inactivation demonstrated motor control from each motor cortex. In contrast, physiological and anatomical interrogation of these pathways support adaptations only in the uninjured hemisphere. Intracortical microstimulation of motor cortex in the uninjured hemisphere of rats with neonatal pyramidotomy produced responses from both forelimbs, while stimulation of the injured hemisphere did not elicit responses from either forelimb. Both anterograde and retrograde tracers were used to label corticofugal pathways. There was no increased plasticity from the injured hemisphere, either from cortex to the red nucleus or the red nucleus to the spinal cord. In contrast, there were very strong CST connections to both halves of the spinal cord from the uninjured motor cortex. Retrograde tracing produced maps of each forelimb within the uninjured hemisphere, and these were partly segregated. This suggests that the uninjured hemisphere may encode separate control of the unimpaired and the impaired forelimbs of rats with neonatal pyramidotomy.

## Introduction

Injury to one hemisphere alters neuronal connections in both the injured and the uninjured hemispheres, especially if the injury occurs early in life. In the case of injury to the corticospinal tract (CST), the principal pathway for voluntary motor control (Porter and Lemon, 1995), descending motor pathways from both hemispheres have been demonstrated to adapt to injury in neonatal rats (Umeda and Funakoshi, 2014) and in humans (Krägeloh-Mann et al., 2017). However, it is not clear which neural circuits from the injured or the uninjured hemispheres are important to mediate motor skill recovery after early brain injury. This conflicting evidence limits the application of therapies designed to strengthen residual neural circuits after unilateral brain injury because we do not know which hemisphere to target.

CST connections between the motor cortex and the ipsilateral spinal cord (ipsiCST) are very strong early in development. Normally, the ipsiCST becomes pruned with postnatal experience to become sparse in maturity (Martin, 2007; Serradj and Martin, 2016). The pathway is much stronger from the uninjured hemisphere after injury to the other hemisphere, if the injury occurs early in development (Staudt, 2010) or if the injury is very large (Staudt, 2010; Umeda et al., 2010). This is due to the lack of competition from the injured hemisphere (Martin, 2007). The ipsiCST is also important for functional recovery after CST injury in adult rats (Carmel et al., 2014; Wahl et al., 2014), monkeys (Sawada et al., 2015), and humans (Cramer et al., 1997; Marshall et al., 2000; Ward et al., 2003).

Recovery of function from neonatal injury of the CST can also be mediated through the rubrospinal tract (RST), which shares many anatomical and functional properties. Both systems may substitute for each other during the execution of skilled movements and collateral sprouting of one tract could contribute to functional recovery when the other one is injured (Kennedy, 1990; Cheney et al., 1991; Han et al., 2015). Neonatal lesion of the CST from one hemisphere led to specific corticorubral and corticopontine plasticity in connections from motor cortex on the injured side (Z’Graggen et al., 2000). The motor cortex that has lost its direct access to the spinal cord via the CST established a bilateral innervation of the red nucleus and the basilar pontine nuclei (Z’Graggen et al., 2000). Recently, it has been reported that the forelimb motor cortex representation was restored and enlarged on the ipsilesional side, and abundant axonal sprouting from the reemerged forelimb area was found in the ipsilesional red nucleus after forced use of the impaired forelimb (Ishida et al., 2016). In addition, blockade of the cortico-rubral tract using a double-viral vector technique caused deficits of the recovered forelimb function (Ishida et al., 2016). Transient inactivation of the red nucleus resulted in abrogated functional recovery in other injury models (Z’Graggen et al., 2000; Siegel et al., 2015; Mosberger et al., 2018). These studies support the red nucleus in the injured hemisphere as an important mediator of recovery after neonatal CST injury.

**Figure 1:**
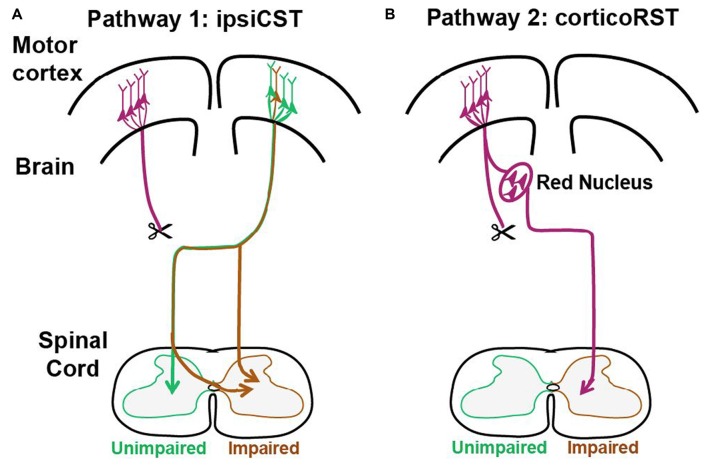
Candidate circuits for recovery of corticospinal tract (CST) function after neonatal pyramidotomy. We created unilateral CST injury by cutting the left CST at the level of the pyramid in the P7 rat. We then examined which spared motor connections adapt to CST injury. **(A)** One candidate spared motor pathway is the CST on the uninjured side of the brain, which has sparse projections to the ipsilateral and impaired side of the spinal cord (ipsiCST). **(B)** Another candidate motor pathway is bypass circuit from the motor cortex on the injured side to the ipsilateral red nucleus; the rubrospinal tract (RST) then crosses to the spinal cord on the impaired side cortico-rubrospinal tract (corticoRST). Scissors showed the cut.

These previous findings support that two pathways from the injured hemisphere or from the uninjured hemisphere may contribute to the function recovery of the impaired forelimbs after early brain injury as shown in Figure 1. The first one is the CST from the uninjured hemisphere that may control the affected forelimb through the ipsiCST (Figure 1A). Alternatively, control may be regained through cortico-rubrospinal tract (corticoRST) from the injured hemisphere to the red nucleus and then to the spinal cord (Figure 1B). While each of these pathways has been implicated in plasticity and functional recovery, they have not been compared against one another in the same study. Thus, it is uncertain which pathway mediates motor control in maturity after neonatal injury. We hypothesized that the ipsiCST would be most effective, based on its strong connections and ability to restore function, even in adulthood. In this study, we found that the function of the impaired forelimbs was controlled from both the injured and the uninjured hemispheres in the adult rats after neonatal pyramidotomy as evidenced by behavioral impairment after inactivation of each motor cortex. We also compared the anatomy and physiology of these pathways through both anterograde and retrograde tracers and intracortical microstimulation (ICMS). We found that large-scale anatomical changes occurred only in the uninjured hemisphere after neonatal pyramidotomy. These findings help to close our gap in understanding about how descending motor connections adapt to CST injury during development.

## Materials and Methods

### Overview

We tested motor control and pathway adaptation after neonatal pyramidotomy using behavioral, anatomical and physiological approaches. For the injury, we used a complete lesion of the CST in the pyramid of the rostral medulla. This lesion ablates the CST from one hemisphere but leaves intact motor cortex connections to the red nucleus on the injured side. The pyramidotomy was performed in rats on postnatal-day 7 (P7), an age at which the developmental stage of the rat brain is histologically similar to that of the newborn human (Palmer et al., 1990), and then rats were reared to maturity. Comparisons were made between the two hypothesized circuits (Figure 1) within the rats with neonatal pyramidotomy and also with uninjured rats to understand the magnitude of adaptation. For the control group, rats did not receive neonatal surgery; they were raised to maturity and then trained and tested on the behavior assays or used for anatomical and electrophysiological testing. Behavioral testing, including pasta manipulation and horizontal ladder walking, was performed when the rats reached adulthood (P80 ± 17) to measure the extent of deficits after neonatal pyramidotomy. In addition, motor cortex inactivation was performed to determine the contribution of motor cortex in each hemisphere to forelimb skill by using the knob supination task in rats (P80 ± 17). For the anatomical analysis, rats (P74 ± 13) received different anterograde tracer injections into each motor cortex, and projections from motor cortex to the red nucleus and the cervical spinal cord were quantified. For retrograde tracing, Fast Blue (FB), was injected unilaterally into the enlargement of the cervical spinal cord, and the number of neurons in motor cortex and red nucleus were quantified. For electrophysiology mapping, adult rats underwent ICMS of the primary motor cortex of both hemispheres. All of the anatomical and behavioral analyses were done by blinded observers. This study was carried out in accordance with the recommendations of National Institutes of Health guidelines, committee of Weill Cornell Medicine. The protocol was approved by the committee of Weill Cornell Medicine.

### General Surgical Methods

Experiments for neonatal pyramidotomy were conducted under anesthesia using 1%–1.5% isoflurane on the P7 Sprague-Dawley rats (weight, 14–19 g). All of the surgeries performed in adult rats–anterograde and retrograde tracing and ICMS–were performed under general anesthesia (80 mg/kg ketamine and 10 mg/kg xylazine, via intraperitoneal injection). Timed pregnant Sprague-Dawley rats were purchased from Charles River Laboratories (Wilmington, MA, USA), and housed in an animal care facility that was dark during the day (9 am–9 pm) and light at night to allow behavioral testing during the dark phase, when rats are most active.

### Animal Number and Justification

A summary of the number of neonatal pyramidotomy and control rats used for each part of the experiment is presented in Table 1. A total 40 rat pups received neonatal pyramidotomy, and only 27 rats with neonatal pyramidotomy were included in the analyses (*n* = 14 for anterograde/retrograde tracing and ICMS; *n* = 13 for behavioral tests) because some rats died and some received incomplete lesions (see the details in Table 1). The number of the control rats was 25 (*n* = 6 for anterograde/retrograde tracing and ICMS; *n* = 19 for behavioral tests). For each analysis, the number of rats used was based on a power analysis of previous studies using alpha of 0.05 and power of 0.8. For the effects of inactivation on the supination task, we powered off of our previous results (Sindhurakar et al., 2017), and determined that a sample size of three was needed in each group; seven rats were used instead since inactivation were performed in both hemispheres. For anterograde anatomical analysis, we used the large effects we had seen previously (Carmel et al., 2013) to determine a sample size of seven in each group. The retrograde analyses were not submitted to power analysis, since the effect sizes were unknown. The group of rats with neonatal pyramidotomy tested on the supination task was separate from the group tested on the ladder and pasta manipulation tasks to avoid any effects of training the reaching forepaw. The rats used for physiology and anatomy were separate from those used for behavior to avoid effects of limb use on anatomy of the motor systems (Maier et al., 2008) or tracer efficiency (Bilgen et al., 2006).

**Table 1:** Numbers of animals with neonatal pyramidotomy (Px).

Experiments	Total rat pups used	Died during or after Px	Died during subsequent surgeries	Incomplete lesion	Over cut	Complete lesion	Used in the study
Tracers/ICMS	40	10	8	7	1	14	14
Pasta/Ladder	10	4	3	0	0	3	6
Supination/Inactivation	10	0	3	4	0	3	7

### Neonatal Pyramidotomy

A unilateral pyramidotomy was performed to transect selectively the CST axons at the level of the rostral medulla oblongata of P7 rats. Using a ventral approach, the left pyramidal tract was exposed by retracting the paratracheal tissue and drilling a small hole on the bone overlying the medullary pyramid. An incision was made into the dura, and the left pyramid was transected using iridectomy scissors marked at the 1 mm desired depth. The skin wound was closed using gluture topical tissue adhesive (WPI, Sarasota, FL, USA), and the pups were warmed on a heating plate until fully awake before being returned to their mother.

### Behavioral Tests

#### Pasta Handling

Adult rats that had received neonatal pyramidotomy (*n* = 6) and uninjured control rats (*n* = 8) were videotaped as they consumed uncooked pieces of vermicelli, and the number of adjustments made with the left and right paw were recorded and analyzed, as previously described (Allred et al., 2008). The number of adjustments made with each forepaw for each of five or more pasta pieces eaten per testing session was averaged.

#### Horizontal Ladder Walking

After the pasta test, rats in the neonatal pyramidotomy group (*n* = 6, the same rats used in the pasta manipulation test) were trained and tested by running across a horizontal ladder with irregularly spaced rungs. A group of control rats (*n* = 6, separate from the control group that performed the pasta manipulation test) were trained and tested only on the ladder walking task. The number of rats chosen for this task is similar to sample size data from a previous study (Carmel et al., 2014) that exhibited large effects. The ladder used is our previously published (Carmel et al., 2010, 2014) adaptation of the original ladder task (Metz and Whishaw, 2002, 2009). During testing sessions, rats were run for 10 trials in each direction on the ladder. All trials were video recorded using a consumer digital video camcorder at 30 Hz with 1200 W of illumination and were analyzed frame by frame. Steps with placement of the palm of the forepaw, between the wrist and the digits, on the rung were scored as good. All other steps were recorded as errors. The overall error rate is the number of errors over the total number of errors and good steps. Baseline error rates were established with at least two testing sessions. In the current study, we only analyzed the forelimbs because it has been shown the pyramidotomy caused deficits of fine forelimb movement (Lee and Lee, 2013). In addition, in adult rats with pyramidotomy, the hindlimb deficits completely resolved (Carmel et al., 2010).

#### Supination Task

In the knob supination task, adult rats with neonatal pyramidotomy (*n* = 7) and control rats (*n* = 5) were placed into a reaching box for reaching, grasping, and supinating a knob to receive a food reward (Meyers et al., 2016; Butensky et al., 2017; Sindhurakar et al., 2017). All rats were trained to supinate with their right forepaw for 3–4 weeks until they were able to turn the knob 60° at a success rate of 70% or more.

#### Motor Cortex Inactivation

To determine which hemisphere controlled the impaired forelimb of adult rats with neonatal pyramidotomy, we used muscimol (Sigma) to transiently inactivate both of right (intact) and the left (injured) motor cortex (Huber et al., 2012; Carmel et al., 2014; Peters et al., 2014). After establishing baseline performance of the control (*n* = 5) and the rats with neonatal pyramidotomy (*n* = 7) on the knob supination task, we implanted a guide cannula over the caudal forelimb area (CFA) of motor cortex, and then an inner cannula was inserted through the guide cannula into cortex (deep 1.5 mm). Each hemisphere received an infusion of 1 μl saline or 0.025 mg/ml muscimol over 5 min. Rats were tested in the supination task at 1 h after infusion. Two days after inactivation, rats were tested again to ensure that muscimol had washed out, and success rates had returned to pre-inactivation values.

### Physiology Tests: ICMS Mapping of Forelimb Motor Cortex

ICMS mapping of motor cortex was performed as previously described, it has been shown that there is a strong correlation between movement and EMG (Brus-Ramer et al., 2009). Briefly, rats were head-fixed in a stereotaxic frame after anesthetization. The torso was supported to enable the forelimbs to hang freely. A craniotomy was made to expose the forelimb motor cortex of both hemispheres. Tungsten microelectrodes (50–100 kΩ; FHC, Bowdoin, ME, USA) were used for stimulation. The electrode was lowered 1.5 mm below the cortical surface, corresponding to layer V of the frontal agranular cortex. At each cortical site (23 sites in total), stimulation with a train of 16 biphasic pulses (0.2 ms in each phase; 333 Hz) was performed, it has shown that a single pulse causes activation of both motor cortex and subcortical structures (Patton and Amassian, 1954; Zappulla et al., 1988), starting at 10 μA and increased in 5 μA steps until a clear response of the right paw or the left paw was detected by visual inspection. The current was lowered until responses were lost; the final stimulation intensity at which a movement was observed in more than 50% of trials defined the movement threshold. If no movement was detected at 100 μA, the site was defined as non-responsive.

### Anatomical Tests

#### Anterograde Tracing

Corticofugal projections were traced using pressure injections of the anterograde tracers biotin-dextran amine (BDA; 10,000 mw, Molecular Probes, Eugene, OR, USA) and Alexa488 dextran amine (10,000 mw, Molecular Probes) into motor cortex as in our previous studies (Carmel et al., 2010, 2013, 2014). BDA had higher sensitivity than Alexa488, so we created two separate groups of rats with neonatal pyramidotomy in order to compare adaptations from each motor cortex: (1) Group I rats with neonatal pyramidotomy had BDA tracing of the injured hemisphere (on the side of the lesioned pyramidal tract); and (2) Group II rats with neonatal pyramidotomy received BDA injection of the uninjured side (hemisphere contralateral to the lesioned pyramidal tract). Control group without neonatal lesion received BDA injection in the left hemisphere. Four injections of 0.5 μl of a 10% BDA or Alexa488 solution were made for each rat in these three groups. The coordinates were as follows: 1.0 mm rostral and 2.5 mm lateral to bregma; 1.0 mm rostral and 3.5 mm lateral to bregma; 2.0 mm rostral and 2.5 mm lateral to bregma; and 2.0 mm rostral and 3.5 mm lateral to bregma. Injections were made in a depth of 1.5 mm below the cortical surface. Each injection was made stereotactically with pulled glass pipettes (50 μm tip diameter) fitted with a 5 μl Hamilton syringe attached to a MicroPump (WPI) at a flow rate of 0.25 μl/min. The pipette remained in place for 2 min after each injection.

#### Retrograde Tracing

Rats in the three groups received injections of retrograde tracer FB (Polysciences Inc., Warrington, PA, USA) into the intermediate zone of the cervical spinal cord gray matter. Under anesthesia, the rat was head fixed, the T1 spinous process clamped, and cervical spinal cord was exposed. The rats in Group I received FB injections into the ipsilateral (to the lesion) side of the spinal cord. The rats in Group II received the injections into the contralateral (to the lesion) side of the spinal cord. Initially, we injected the anterograde tracer BDA and the retrograde tracer FB at the same time, but there was significant mortality. Therefore, we changed the surgical procedure to inject BDA into cortex first, and then we injected FB into the spinal cord 1 week later. The intact rats in the Control group received injections into the left side of the spinal cord after the anterograde tracer injection. Two-hundred nanoliters of 2% FB was injected through pulled glass pipettes (tip diameter <50 μm) into each of three spinal levels: C5, C6, and C7 segments injections were made at the edge of the dorsal column, 0.8–1 mm lateral from the spinal cord midline, and at a depth of 1 mm from the dorsal surface). The micropipette remained in place for 5 min after each injection to avoid backflow of the tracer.

#### Perfusion and Tissue Preparation

Fourteen days after FB tracing, rats were perfused transcardially with 0.1 M phosphate buffered saline (PBS) containing 10,000 IU/L of heparin followed by 4% paraformaldehyde in 0.1 M phosphate buffer. The brain and the cervical spinal cord were removed, post-fixed overnight, and then transferred to a solution of 30% sucrose in 0.1 M phosphate buffer for 3 days. The brain was blocked into three parts: one part including the primary motor cortex from bregma 4 to −2, one including the red nucleus from bregma −5 to −7, and one including the lesion site in the rostral medulla. The brain blocks and the cervical spinal cord were frozen with dry ice and kept at −80°C until sectioning. Coronal sections (40 μm) were cut on a cryostat.

#### Histochemistry

Biotin-avidin histochemistry was used to visualize BDA-labeled axons in the red nucleus and the cervical spinal cord. In brief, the sections were: (1) washed with 0.1 M PBS for 30 min; (2) incubated in 0.3% hydrogen peroxide for 10 min to quench endogenous peroxidase; (3) washed with 0.1 M PBS for 30 min; (4) incubated with an avidin-biotin-peroxidase complex (ABC kit, Vector Labs, Burlingame, CA, USA); and (5) visualized with 3,3′-diaminobenzidine and nickel intensification (SK4100, Vector Labs). Brain sections containing the red nucleus were counterstained with 0.1% of cresyl violet to identify the boundaries of the nucleus.

**Figure 2:**
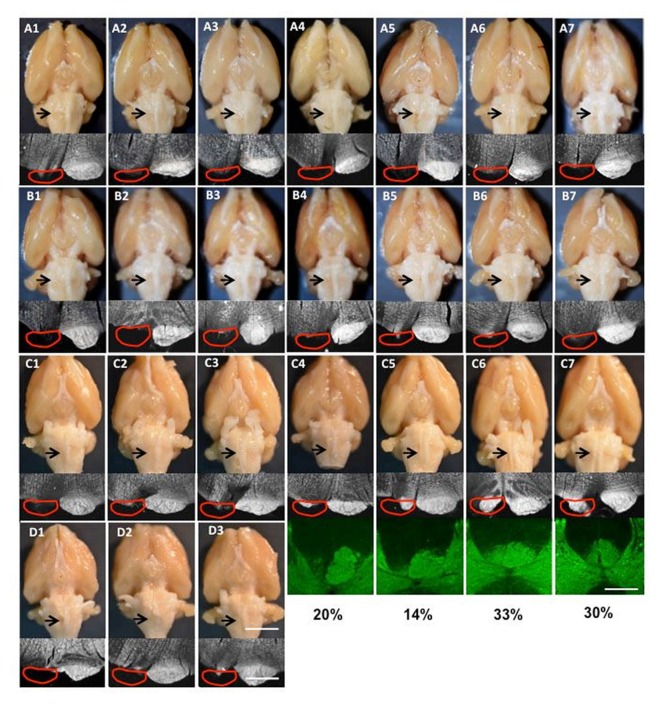
Images of lesions of all rats used in the study. **(A1–7)** Group I for tracing and intracortical microstimulation (ICMS); **(B1–7)** Group II only for tracing; **(C1–7)** supination task and motor cortex inactivation; **(D1–3)** pasta manipulation and ladder walking. The top row is the ventral view of the whole brain; the arrow shows the cut lesion. The second row shows dark-field images of coronal section through the cut level. The red line shows the approximate position of the pyramid that was the cut. The bottom rows in **(C4–7)** show protein kinase C-γ (PKC-γ) staining in the dorsal column with the percentage of CST spared after neonatal pyramidotomy. Bar = 4 mm (top row in **D3**), 12.5 mm (bottom row in **D3**), 5 mm (the third row in **C7**).

Protein kinase C-gamma (PKC-γ, which labels CST axons within the tract) immunostaining was used to evaluate lesion completeness in rats with possible CST sparing after pyramidotomy (Starkey et al., 2005). Briefly, free-floating sections were incubated with a rabbit anti-PKC-γ antibody (1:500, Santa Cruz Biotechnology, Dallas, TX, USA) overnight at 4°C and with a donkey anti-rabbit Alex488 antibody (1:800, Molecular Probes) for 1.5 h at room temperature. The sections were then coverslipped with VECTASHIELD Antifade-Mounting Medium (Vector Labs) and imaged with a fluorescence microscope (ECLIPSE *Ni*, Nikon, Tokyo, Japan). All rats involved in the anatomical experiments (Figures 2A,B) also had confirmation of complete lesions by inspecting the spinal cord for BDA (Group I) or Alexa488 dextran amine (Group II); none was found (results not shown).

### Neuroanatomical Analyses

#### Determining the Extent of Pyramidotomy Lesions

As shown in Figure 2, for each rat the pyramidal tract lesion site was examined macroscopically and microscopically with darkfield imaging of the location and completeness of the lesion as well as for damage to adjacent structures. For rats with lesions that were not clearly complete, PKC-γ staining of the cervical spinal cord was performed. In cases the lesion was not complete, the optical density of PKC-γ immunostaining for each main CST (at the base of the dorsal columns) was performed. The percent sparing was computed as the ratio of the optical density of the partially lesioned CST divided by the intact CST.

#### Distribution of Fast Blue Injections in the Spinal Cord

Different regions of cortex terminate in different laminae of the spinal cord, so understanding the distribution of retrograde tracer in the spinal cord is important (Martin, 1996; Dum and Strick, 2002). In order to confirm that the retrograde tracer FB is distributed within the C5, C6, and C7 the same way across all rats, we used image processing and statistical analysis. First, spinal cord slices of the C5, C6, and C7 were imaged and registered to respective ideal images (Watson et al., 2009) using the warp function in MATLAB. The fluorescence images for each group of rats (control, tracing ipsilateral to pyramidotomy, and tracing contralateral to pyramidotomy) were then used for quantitative analysis. For each cervical level, we performed a permutation test to determine if there were significant differences between the retrograde tracer distributions.

#### Quantification of Anterograde Tracing

Axon length was measured using the “space balls” probe in the Stereo Investigator stereology program (MBF Bioscience), as in our previous studies (Brus-Ramer et al., 2007; Carmel et al., 2010, 2013, 2014). After we traced the areas of interest, the red nucleus and the spinal cord gray matter in the three groups, the program places virtual spheres (“space balls”) within the tissue. At 1000× magnification, we marked intersections between the virtual sphere and labeled axons within the gray matter. Axon length estimates were made from five (the red nucleus) or two (the spinal cord) sections for each rat and the lengths averaged. To control for the efficiency of anterograde tracing, the axon lengths within the red nucleus and spinal cord was normalized to the number of axons within the cerebral peduncle and the main tract of the spinal cord, respectively, as we have done previously (Carmel et al., 2010, 2013, 2014). After counting the BDA-labeled axons in the spinal cord, the interactions between the space ball probes and the axons were used for analysis of axon distribution. We performed a permutation test to determine if there were significant differences in the BDA-labeled axon distributions in the spinal cord of the control and injured rats.

#### Quantification of Retrograde Tracing

For counting the FB-labeled neurons in the primary cortex, motor cortex was cut coronally, and every tenth section was collected from bregma +4 mm to −2 mm (14 total). The cortex and internal anatomical landmarks such as anterior commissure and ventricles were traced using NeuroLucida software (MBF Bioscience, Williston, VT, USA). All the FB-labeled neurons in these 14 sections were counted at 200× magnification and then reconstructed using NeuroLucida. For counting the FB-labeled neurons in red nucleus, every sixth section was collected from the brain block including red nucleus from bregma −5 to −7 (5 total). Among five sections, the most rostral one has only parvocellular part, the most caudal one has only magnocellular part, and the other three have both parts. The left and right red nucleus in all five sections was traced using NeuroLucida, respectively. All the FB-labeled neurons in these five sections were counted at 20× objective using NeuroLucida.

#### Quantification of Neuron Density and Spatial Distributions

In order to combine results from rats within a group, and to compare results across the rat groups, the retrogradely traced neurons within motor cortex were normalized to a common coordinate axis. For this we co-registered all slices to the corresponding slices from a template rat brain atlas; this is similar to the procedures for human brain imaging (Brett et al., 2002) and helps to normalize differences due to inter-animal variability in brain size and shape and especially differences in tissue sectioning. We opted to use a 3-dimensional rat-brain model called Atlas 3D (Hjornevik et al., 2007) that has been reconstructed from the Paxinos and Watson’s (2005) anatomical rat brain atlas. This 3D model allows the matching of slices that may have some angular displacements and or do not match the standard atlas slice positions. As described in the quantification of retrograde tracing section above, the histological slices *s*_x_ (*x* = 1–14) were made at specific stereotactic distances from bregma. For the purpose of co-registration, first each *s*_x_ slice was positioned so that the cortical surface of the histological section aligned with the 3D model. At this location, the *s*_x_ was manually translated, scaled and rotated to best fit the template brain slice at that position and angle. The corresponding transformation matrix for this co-registration was acquired and applied on the traced neuron coordinates of that slice. This allowed the projection of all the traced neurons onto a common coordinate axis. The neuron count was then obtained from these transformed neuron traces.

### Statistical Analysis

#### *T*-Tests for Behavioral and Anatomical Comparisons

Unpaired *t*-tests were used to analyze data from pasta manipulation and horizontal ladder walking tasks. It was also used to analyze differences in number of BDA labeled axons in the dorsal column, length of BDA labeled axons in the spinal cord, and number of FB labeled neurons in the cortex. *Post hoc* analyses for supination task data were analyzed using both one- and two-tailed paired *t*-tests. For all analyses with unequal sample sizes and variance, the Welch’s *t*-test was used.

#### Permutation Tests for Distributions

We used these statistical tests to determine distribution changes in ICMS motor mapping and the distribution of retrogradely labeled neurons in motor cortex. For the chosen statistic used 1000 permutations and set the significance threshold at 95%. For ICMS mapping, the statistic used was the euclidean distance between group means. For analyzing differences between groups in distribution of retrograde tracers injected into the spinal cord we used a statistic that was the sum of euclidean distances between mean group images. For analyzing differences in density maps of the FB-labeled neurons from rat groups (contralateral vs. ipsilateral to the injection of FB in rats with and without injury), we used the Kullback-Leibler Divergence statistic (Kullback and Leibler, 1951). For multivariate data that was assumed to have normal distributions, we used the distance between means statistic for permutation testing. For non-parametric data, we used the Kullback-Leibler divergence statistic that is independent of the shape of the distribution.

In order to assess the injury related changes in the spatial and density distributions of the neuron maps, we estimated the probability density distributions for the contralateral and ipsilateral (to the injection of FB) neuron maps in the control and the injured rat groups. We used this method to test the hypothesis that injury could lead to a shift of neuron density centers. For density estimation we make use of an unsupervised machine learning technique called Gaussian Mixture Model (GMM), which is a multivariate nonparametric method that uses an Expectation-Maximization learning algorithm to search for the optimal data density centers whose linear combination forms the measured data density mixture (Redner and Walker, 1984; Bishop, 2006). GMM had an advantage that it blindly searches for underlying separable data density centers, without requiring *a priori* knowledge about the data distribution. Assigning the neurons with *a priori* labels based on anatomical structures, such as the rostral or CFA (Umeda et al., 2010), could have produced bias in the analysis. These probability distribution density maps were then averaged across rats for each group, generating one density distribution map per group. It should be noted that each rat’s neuron density was represented as a probability density map before averaging across rats. This obviates the need to normalize by the absolute number of neuron count when combining the data across rats.

#### ANOVA for Comparison of 3 Groups

One-way analysis of variance (ANOVA) was used to analyze the differences between the three groups (Control, Group I and Group II) in respect to the BDA labeled axons in the peduncle, BDA labeled parvocellular/magnocellular axons in the red nucleus, and FB labeled neurons in the red nucleus. One-way repeated measures ANOVA tests were used to identify differences in supination performance behavioral data before and after motor cortex inactivation.

#### Controlling for Multiple Comparisons

Bonferroni correction was performed on multiple measures performed in the same group of ratss. The ladder walking and pasta manipulation tasks were performed on two different groups of rats, and both forelimbs were tested. So the *p*-values were corrected for two comparisons for each task. For the inactivations, ANOVA was followed by Bonferroni-corrected *post hoc* testing. For anatomical data, anterograde tracing was done at two levels of the red nucleus (parvocellular and magnocellular) and the spinal cord. In addition, both left and right sides were analyzed. So the *p*-values were corrected for six comparisons. For the retrograde tracing, FB-labeled neurons were counted in red nucleus and motor cortex on each side, so the *p*-values were corrected for four comparisons.

## Results

### Pyramid Lesions Without Incursion Into the Underlying Medulla in All Rats Included in the Study

For all rats that received neonatal pyramidotomy, the pyramid lesion was inspected after perfusion as an adult, both by gross examination and by histological verification. For anatomical (tracers) and physiological (ICMS) analysis, 14 rats with complete lesions were used (Table 1). The lesion was confirmed in the spinal cord by injection (ipsilateral to the lesion) with the anterograde tracer BDA or Alex488. The brains of the 14 rats with complete lesions were shown in the Figures 2A,B. For pasta and ladder tests, six rats with neonatal pyramidotomy were used, including three rats that had complete lesions confirmed (Table 1, Figure 2D) and three rats whose lesions were not examined because they died in a subsequent procedure. For inactivation tests, seven rats with neonatal pyramidotomy were used, including three rats with complete lesion and four rats with incomplete lesion (Table 1, Figure 2C). The percentage of the dorsal column with PKC-γ staining in the four rats with incomplete lesion was shown in Figure 2C.

### Persistent Motor Deficits in Rats With Neonatal Pyramidotomy

**Figure 3:**
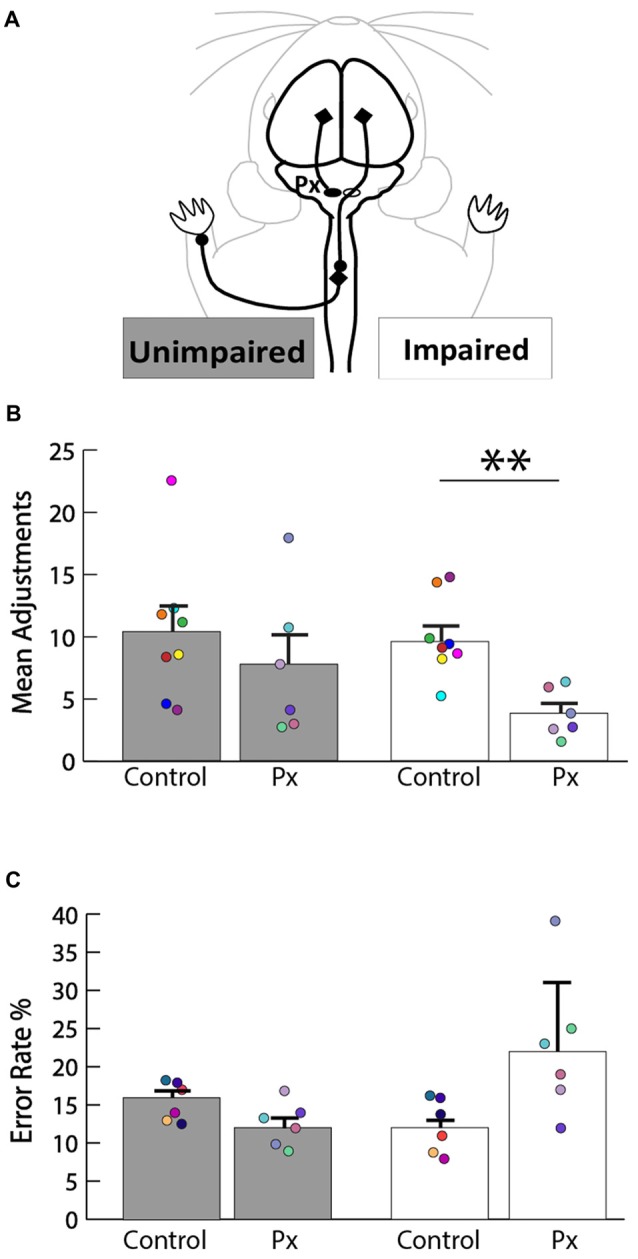
Motor deficits in rats with neonatal pyramidotomy. **(A)** Schematic of the intact CST that contributes to function of the unimpaired (left forepaw) and the impaired (right forepaw) forelimbs. **(B)** Pasta manipulation. The rats with neonatal pyramidotomy (Px) made fewer adjustments with their right forepaw than the control rats. No significant differences were detected in the amount of adjustments made by the left forepaw between the control and the Px rats. **(C)** Ladder walking. The right forepaw of the Px rats had a greater error rate percentage compared to that of the controls; however, left forepaw placement error was similar in the control and the Px rats. In addition, there were greater errors made with the right forepaw than the left forepaw in the Px rats. Data = mean ± SEM. Plotted dots represent each rat’s behavioral data points in each group. Each rat’s data are identified by the color of the dot. **p < 0.01.

We sought to determine the extent of the motor deficits in adult rats caused by the neonatal pyramidotomy. The pasta manipulation task (Figure 3A) was performed in order to assess differences in forepaw use. Since pyramidotomy was performed on the left, rats were predicted to have more fewer right than left forepaw manipulation. Indeed, rats with neonatal pyramidotomy made more adjustments with their left (unimpaired) forepaw than their right (impaired) forepaw (Figure 3B). In contrast, control rats made equal adjustments with their left and right forepaws. Rats with neonatal pyramidotomy made significantly fewer adjustments with their right (impaired) forepaw than the control rats; *t*_(12)_ = 3.59, Bonferroni-adjusted *p* = 0.008, *d* = 2.02, power = 0.93. The number of adjustments made with the left (unimpaired) forepaw was not significantly different between the two groups; *t*_(12)_ = 0.84, Bonferroni-adjusted *p* = 0.84. On the horizontal ladder walking task (Figure 3C), rats with neonatal pyramidotomy show a trend towards higher error rates of right (impaired) forepaw placement compared with uninjured controls, however this was not significant; *t*_(10)_ = 2.48, Bonferroni-adjusted *p* = 0.06. There was no significant difference in left (unimpaired) forepaw error rate between the neonatal pyramidotomy group and the control group; *t*_(10)_ = −2.14, Bonferroni-adjusted *p* = 0.12. Together, these data indicate that neonatal pyramidotomy produces motor deficits that persist into adulthood.

### Forelimb Motor Control From Both Hemispheres in Adult Rats With Neonatal Pyramidotomy

**Figure 4:**
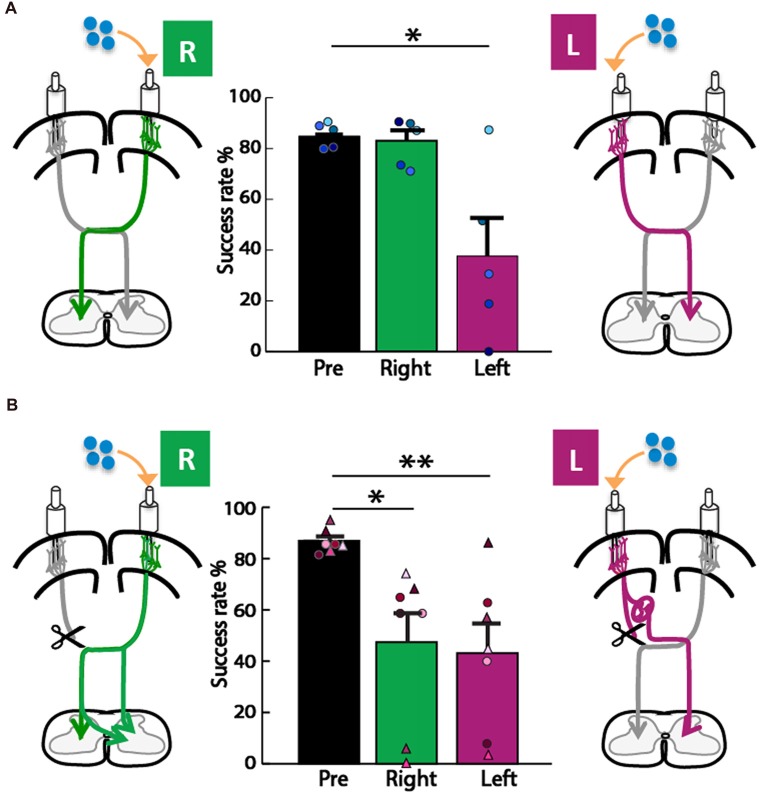
Forelimb motor control from both hemispheres in adult rats with neonatal pyramidotomy. **(A)** Control group. Muscimol, shown in blue, was injected into the left (L) or right (R) motor cortex. In the control rats, there was no significant difference between baseline performance (black, pre) and performance (green, right) after right motor cortex inactivation. Performance after left motor cortex inactivation (magenta, left) was significantly reduced compared to baseline performance (black, pre). Plotted dots in different shades of blue represent each rat’s success rate data points. **(B)** Neonatal pyramidotomy. Compared to baseline performance (black, pre), rats with neonatal pyramidotomy showed deficits in performance after both right (green, right) and left motor cortex (magenta, left) inactivation. In addition, rats with neonatal pyramidotomy showed larger impairments on the supination task than the controls after receiving right motor cortex inactivation. Plotted dots and triangles in different shades of pink represent each rat’s success rate data points. Dots in shades of pink indicate complete pyramidotomy and triangles represent rats with incomplete pyramidotomy. *p < 0.05, **p < 0.01.

We next asked whether the motor cortex in the left or right hemisphere exerts control of the impaired forepaw, since each motor cortex is the origin of one of the two pathways for motor control we considered (Figure 1). To assay forepaw control, we used the knob supination task (Meyers et al., 2016; Butensky et al., 2017; Sindhurakar et al., 2017); which was titrated to the maximum level achievable by rats with neonatal pyramidotomy (60° from pronation to supination). We then titrated the concentration of muscimol that was used for cortical inactivation to cause a significant deficit in the contralateral forelimb in uninjured adult rats. We tested what percent of trials rats could turn the knob 60° in supination, a movement lost with pyramidotomy (Carmel et al., 2013; Sindhurakar et al., 2017). In the control animal group (Figure 4A), the overall ANOVA was significant *F*_(2,8)_ = 10.72, *p* = 0.005, *f* = 1.64, power = 0.93. *Post hoc* analyses were done to test if there was a decline in performance. A decrease was observed after left hemisphere inactivation (Bonferroni-adjusted *p* = 0.03, *d* = 1.61, power = 0.77), but not after right hemisphere inactivation (Bonferroni-adjusted *p* = 0.86). In the neonatal pyramidotomy group (Figure 4B), the overall ANOVA was significant also; *F*_(2,12)_ = 8.48, *p* = 0.005, *f* = 1.89, power = 0.91. *Post hoc* analyses revealed a significant decline in performance following both left (Bonferroni-adjusted *p* = 0.008, *d* = 1.63, power = 0.95) and right (Bonferroni-adjusted *p* = 0.03, *d* = 1.22, power = 0.77) hemisphere inactivation. There was no difference between inactivation of the left or right motor cortex (Bonferroni-adjusted *p* = 1.00). If only the rats with complete pyramidotomy were included in this analysis, the overall ANOVA was also significant (*F*_(2,4)_ = 7.90, *p* = 0.04, *f* = 1.99). *Post hoc* testing showed a significant effect for the right motor cortex inactivation (Bonferroni *p* = 0.02, *d* = 6.94) but not the left (Bonferroni *p* = 0.26).

### Anatomy of Motor Pathways in Rats With and Without Neonatal Pyramidotomy

**Figure 5:**
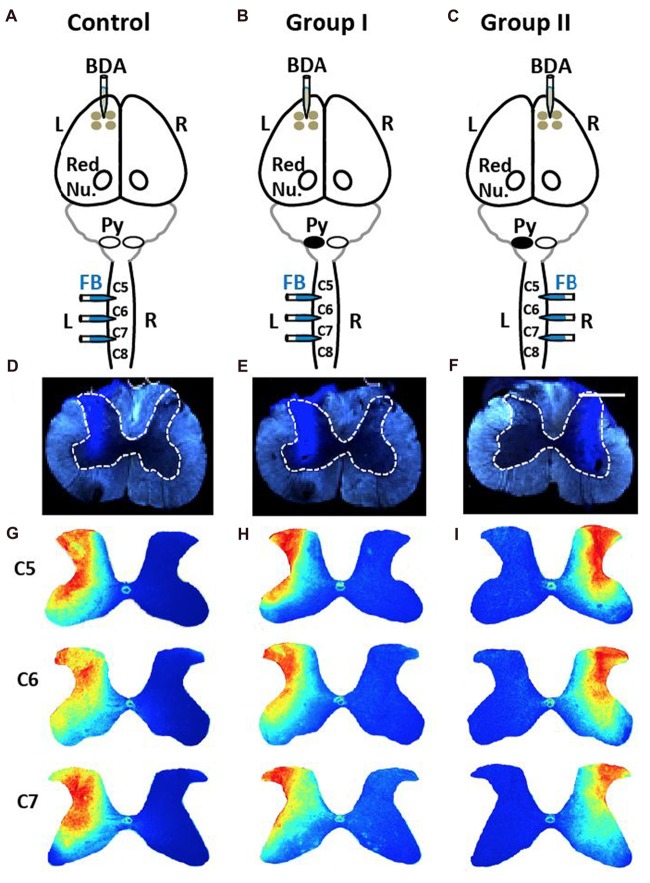
No differences in injections of Fast Blue (FB) into spinal cord among three groups. **(A)** Control rats received four injections of the anterograde tracer biotin-dextran (BDA) into the left (L) primary cortex and three injections of the retrograde tracer FB into the left side of the spinal cord. Red Nu., red nucleus. **(B)** Group I rats with neonatal pyramidotomy (indicated by the black oval next to pyramid, Py) received BDA injections into the left primary cortex and the FB injections into the left side of the spinal cord. **(C)** Group II rats with neonatal pyramidotomy received BDA injections into the right (R) primary cortex and the FB injections into the right side of the spinal cord. **(D–F)** Representative images of FB injections in the control rats and the rats with pyramidotomy. The dotted lines show the gray matter in the spinal cord. Bar = 0.5 mm. **(G–I)** Heat maps of distribution of FB in the spinal cord of C5, C6 and C7 of the control rats and the rats with neonatal pyramidotomy. Note the similar distribution among three groups.

We combined retrograde injections of spinal cord (FB) and anterograde labeling of motor cortex (BDA) in uninjured control rats (Figure 5A) and rats with neonatal pyramidotomy. To compare the two pathways in neonatal rats, we employed two groups of rats (Figures 5B,C) that had labeling of each of the proposed pathways for motor control (Figure 1). This was necessary because we did not find anterograde or retrograde tracers that were as efficient as BDA and FB. The retrograde injections had to meet two criteria: (1) limited to one half of the spinal cord; and (2) the distribution of tracer within the spinal cord gray matter must be similar across rats. As shown in the images in Figures 5D–F the FB injections were limited to one half of the spinal cord. We used image processing and statistical analysis to compare the distributions of the FB labeling in the spinal cord. The fluorescence images for individual rat in each group (Control, Figure 5D, Group I, Figure 5E, Group II, Figure 5F) were used to create a heatmap of the group distribution (Figures 5G–I) and quantitative analyses. There are no significant differences across groups for all three cervical levels that FB was injected (Bonferroni-corrected *p* = 1.0, 0.57 and 0.48 for C5, C6 and C7, respectively).

### Cortical Maps Show Large Increase in Ipsilateral Representation, Especially in the Rostral Forelimb Area, and Loss of Contralateral Representation

**Figure 6:**
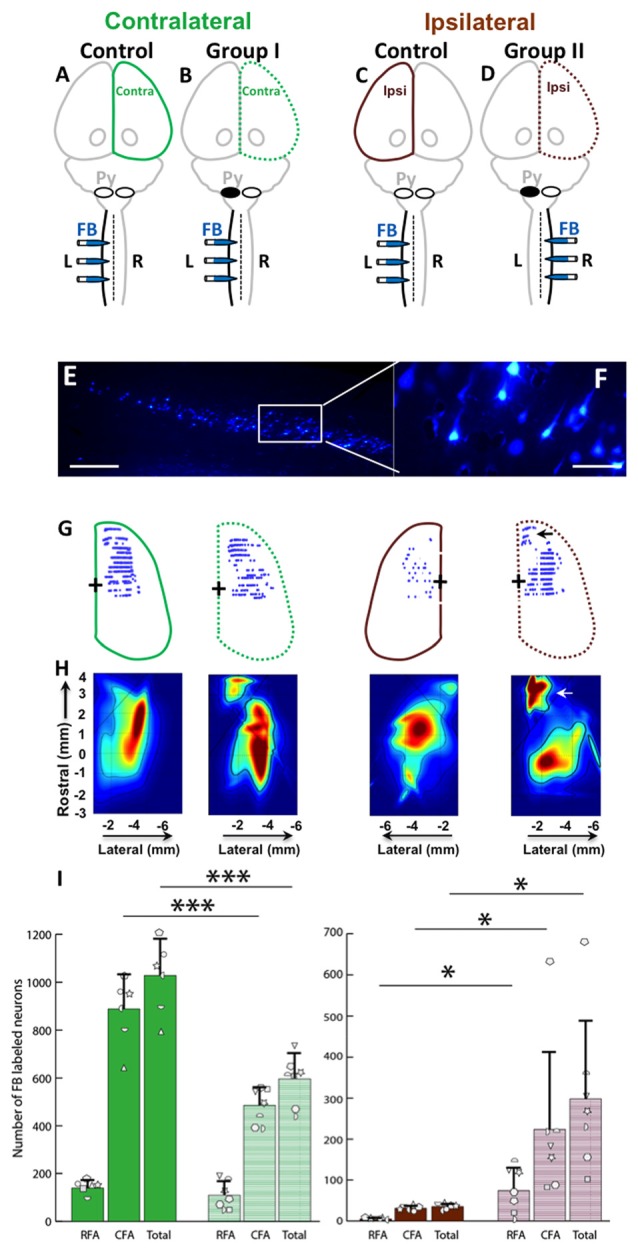
Cortical maps showed large increase in ipsilateral representation, especially in the rostral forelimb area (RFA), and loss of contralateral representation. **(A–D)** Experimental schema of injections of FB, pyramid (Py) cut, and shading of the cortex that was analyzed. **(A**,**C)** Control rat with FB injection into the left (L) side of the spinal cord; this was one group of rats, but each hemisphere was compared against a different group (Group I and Group II) of the rats with neonatal pyramitomy. The contralateral (Contra) hemisphere was compared against Group I, and the ipsilateral (Ipsi) hemisphere was compared against Group II. **(B)** Group I rats with FB injection into the left (unimpaired) side of the spinal cord. **(D)** Group II rats with FB injection into the right (impaired) side of the spinal cord. **(E,F)** FB-labeled neurons in the motor cortex (1.5 mm rostral to bregma) of a representative rat from Group II. Bar = 250 μm **(E)** or 50 μm **(F)**. **(G)** Maps of distribution of the FB-labeled neurons in the cortex of the control rats and the rats with neonatal pyramidotomy. The + symbol indicates bregma. **(H)** Heat maps of probability density distribution of the FB-labeled neurons in the cortex of the control rats and the rats with neonatal pyramidotomy. Dark red indicates high probability (approach 1) and dark blue indicates low probability (approaching 0) There were two distinct representations in the rats with neonatal pyramidotomy, the (RFA, indicated by the arrows) and the caudal forelimb area (CFA) in the ipsilateral cortex. **(I)** The number of the FB-labeled neurons in the cortex. Note that the FB-labeled neurons in the ipsilateral cortex were significantly increased in the rats with neonatal pyramidotomy in comparison to the control rats; however, the numbers in the contralateral cortex were significantly reduced. Plotted white shapes represent the number for each rat in each group. Each rat’s data corresponds to a type and orientation of shape. Data = mean ± SD. *p < 0.05, ***p < 0.005, significant compared to the corresponding values in the control rats.

FB was injected into the spinal cord of Control, Group I and Group II as shown in Figures 6A–D. Then, the retrogradely labeled neurons in the cortex (Figures 6E,F) were counted. We were primarily interested to understand if differences exist between the neonatal pyramidotomy and control groups in the number and distribution of retrogradely labeled neurons in the contralateral (to the injection of FB) hemisphere (Group I vs. Control rats; Figures 6A,B), and in the ipsilateral (to the injection of FB) hemisphere (Group II vs. Control rats; Figures 6C,D). We observed a statistically significant difference between the ratio of contralateral vs. ipsilateral neurons in the control and the neonatal pyramidotomy rats. In the ipsilateral hemisphere, the total number of retrogradely labeled neurons were significantly larger in the rats with neonatal pyramidotomy in comparison to the control rats (*t*_(6.02)_ = 3.67, Bonferroni-adjusted *p* = 0.04, *d* = 1.96, power = 0.89). On the other hand, in the contralateral hemisphere, the total retrogradely labeled neurons were fewer in the rats with neonatal pyramidotomy as compared to the control rats (*t*_(11)_ = −6.08, Bonferroni-adjusted *p* = 0.0003, *d* = 3.32, power = 1.00; Figures 6G–I). We performed a secondary analysis to investigate the number of FB labeled neurons in the caudal and rostral forelimb area (RFA) of both the ipsilateral and contralateral cortex. Neonatal pyramidotomy rats showed greater amounts of labeled neurons in the RFA and CFA of the ipsilateral cortex compared to controls, they separately showed a significant increase in the neuron count (RFA: *t*_(6.029)_ = 3.39, *p* = 0.02, *d* = 1.81 power = 0.84, CFA: *t*_(11)_ = 2.49, *p* = 0.03, *d* = 1.45, power = 0.66). The corresponding decrease in CFA was found to be significant (*t*_(11)_ = −6.52, *p* = 0.00004, *d* = 3.53, power = 1.00) but not in RFA; (*t*_(11)_ = −1.12, *p* = 0.29).

In addition to comparing the number of neurons in each area, we compared the overall distribution of neurons in the same coordinate space. We observed a statistically significant difference in the probability density of the neurons in the ipsilateral hemisphere of the control compared to the rats with neonatal pyramidotomy (Kullback-Leibler divergence, *p* < 0.001), with an enhanced and markedly segregated density distribution centered in the RFA of their ipsilateral hemisphere (see Figure 6H). There was also a significant difference between the contralateral hemispheres of the controls vs. the rats with neonatal pyramidotomy (*p* < 0.001). Thus, there are marked changes in the representation of both the ipsilateral and contralateral forelimbs in rats with neonatal pyramidotomy. In addition, the RFA may preferentially connect to the ipsilateral spinal cord in these rats.

### Spinal Axons Are Dense for the ipsiCST in Rats With Neonatal Pyramidotomy

**Figure 7:**
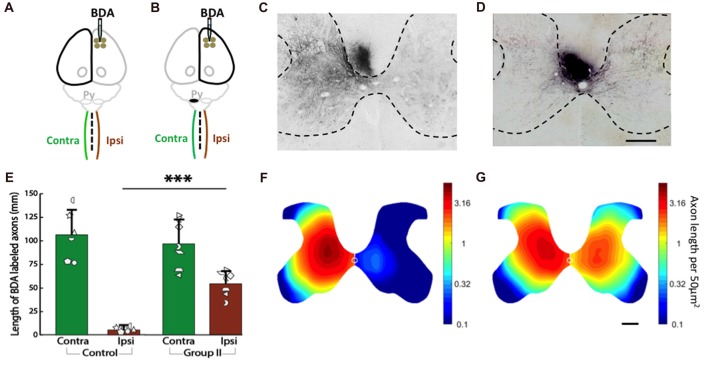
Strong bilateral connections from the motor cortex of the uninjured hemisphere after neonatal pyramidotomy. **(A,B)** Experimental schema of injections of BDA, pyramid (Py) cut, and the analysis of the spinal cords in the Control **(A)** and Group II **(B)**. **(C,D)** BDA-labeled axons in the spinal cords of the Control **(C)** and Group II **(D)**. Bar = 250 μm. **(E)** Average BDA-labeled axon length in the spinal cords of the Control and the Group II. Note that there were significant differences in the distribution and the length of the BDA-labeled axons in the ipsilateral side, but not in the contralateral side of the spinal cord between the Control and the Group II. Plotted white shapes represent each rat’s data points. Each rat’s data corresponds to a type and orientation of shape. **(F,G)** Heat maps of probability density distribution of the BDA-labeled axons in the spinal cords of the Control **(F)** and the Group II **(G)**. Note that the scale is logarithmic. In Group I, the pyramidotomy removed all of the CST axons from the injured hemisphere; therefore, Group I was not shown here. ***p < 0.005, significant compared to the same side of the Control.

We measured corticospinal axon distribution and length in the gray matter of each half of the C6 spinal cord 2 weeks after injection of BDA into cortex of the uninjured control rats (Figure 7A) and the rats with neonatal pyramidotomy (Figure 7B). In our previous work (Carmel et al., 2010, 2013, 2014) and in others (Tan et al., 2012), we have observed that axon termination density and distribution at C6 is representative of the other levels of the cervical enlargement. The BDA-positive axons in the gray matter of both sides of the spinal cord were counted, and then the distribution were analyzed for the control (Figures 7C,E) and the rats with neonatal pyramidotomy (Figures 7D,F). As shown in Figure 7C, in the control rats, BDA-positive axons were mainly detected in the contralateral side (to the injection of BDA) of the spinal cord. On the other hand, both the contralateral and the ipsilateral (to the injection of BDA) sides of the spinal cord of the injured rats had dense innervation with BDA-positive axons (Figure 7D). These results are quantitated in Figure 7E. While corticospinal axon length was not different in the contralateral (to the injection of BDA) side of the spinal cord between the control and the rats with neonatal pyramidotomy (*t*_(11)_ = 0.53, Bonferroni-adjusted *p* = 1.00), the axon length on the ipsilateral (to the injection of BDA) side of the spinal cord was much higher in injured rat than that in the control; *t*_(6.27)_ = −9.81, Bonferroni-adjusted *p* = 0.0003, *d* = 5.25, power = 1.00 (Figures 7E,F).

Finally, we compared the distribution of axons as represented by the heat maps of axon density in Figures 7F,G (note that the scale is logarithmic). The distribution of axons on the contralateral (to the injection of BDA) side of the spinal cord was not different between the control and the injured rats (*p* = 0.45). However, the distribution of axons was significantly different on the ipsilateral (to the injection of BDA) side of the spinal cord between the control and injured rats (*p* = 0.0001). In addition, there was no significant difference between the contralateral and the ipsilateral (to the injection of BDA) sides of the spinal cord of the injured rats (*p* = 0.10) while significant difference was detected between the contralateral and the ipsilateral (to the injection of BDA) sides of the spinal cord of the control rats (*p* = 0.003). These distribution analyses suggest that the axons in the ipsilateral (to the injection of BDA) spinal cord of rats with neonatal pyramidotomy are distributed more similarly to the contralateral (to the injection of BDA) projection of the control rats.

### No Outgrowth of the corticoRST in the Injured Hemisphere

**Figure 8:**
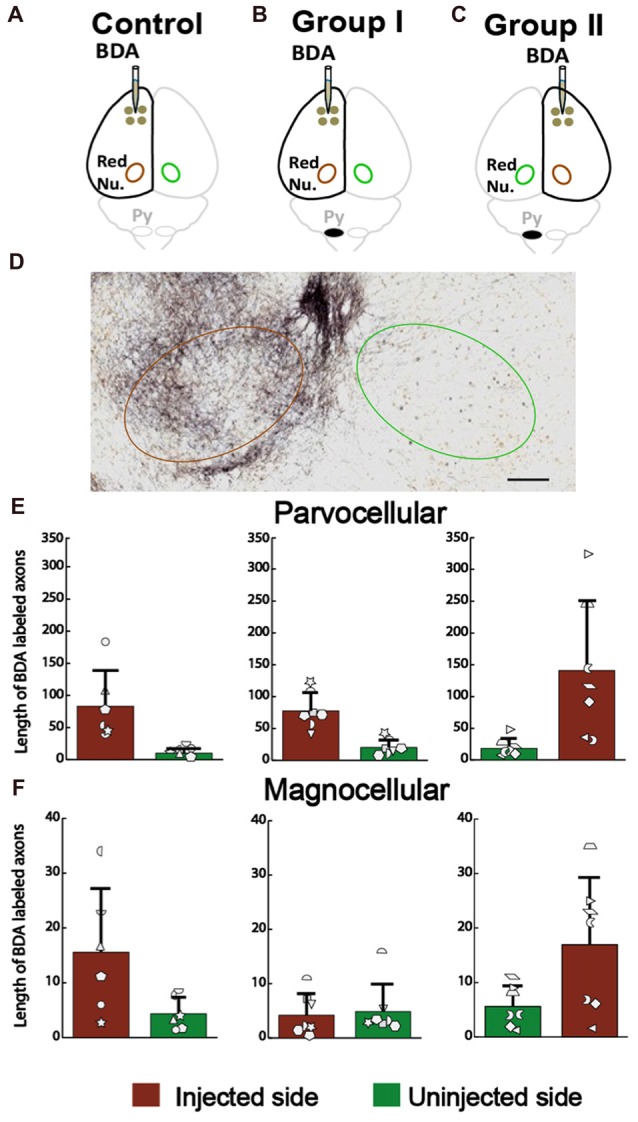
No increase in the corticorubral axons from the injured hemisphere after neonatal pyramidotomy. **(A–C)** Experimental schema of injections of BDA, pyramid (Py) cut, and the analyzed sides of the red nucleus (Red Nu.) in the Control **(A)**, Group I **(B)** and Group II **(C)**. **(D)** The BDA-labeled axons in the red nucleus. Brown circle shows the BDA injected side; the green one the uninjected side. Bar = 125 μm. **(E)** Average BDA-labeled axon length in parvocellular red nucleus. There were no differences in the injected or in the uninjected side of the red nucleus among three groups. **(F)** Average BDA-labeled axon length in magnocellular red nucleus. There were no differences in the injected or in the uninjected side of the red nucleus among three groups. **(E,F)** Plotted white shapes correspond to each rat’s data points. Each rat’s data in the Control group, Group I and Group II were represented by a particular shape.

For the corticoRST, we analyzed anterograde connections from the motor cortex to the red nucleus, since that was our hypothesized locus of plasticity within the corticoRST. If this is a pathway for recovery, we expect more axon length in the red nucleus on the side of pyramidotomy. The anterograde tracer BDA was injected into the left cortex of the control (Figure 8A) and the rats with neonatal pyramidotomy (Group I, Figure 8B), and the right cortex of the rats with neonatal pyramidotomy (Group II, Figure 8C). The BDA-positive axons in the parvocellular and the magnocellular portions of the red nucleus (Figure 8D) were counted for the control and the rats with neonatal pyramidotomy. The results showed no differences in the corticorubral connections in the parvocellular red nucleus between the injected or uninjected sides (crossed axons) of the control and the rats with neonatal pyramidotomy; injected side *F*_(2,17)_ = 1.54, Bonferroni-adjusted *p* = 1.00, uninjected side *F*_(2,17)_ = 1.33, Bonferroni-adjusted *p* = 1.00 (Figure 8E). There was also no difference in the magnocellular red nucleus *F*_(2,17)_ = 3.43, Bonferroni-adjusted *p* = 0.34, uninjected side *F*_(2,17)_ = 0.15, Bonferroni-adjusted *p* = 1.00 (Figure 8F). Therefore, these results showed that there was no outgrowth in the corticorubral connections that might mediate recovery in the corticoRST.

**Figure 9:**
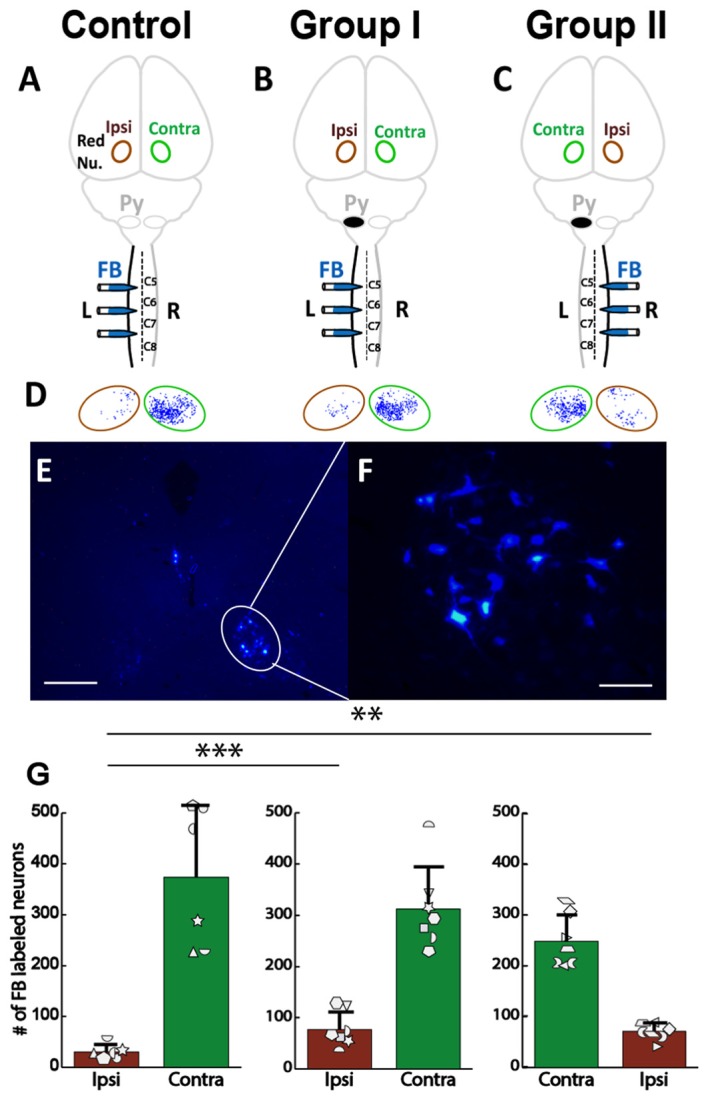
No increase in rubrospinal connections in the corticoRST circuit. **(A–C)** Experimental schema of injections of FB, pyramid (Py) cut, and analysis of the red nucleus (Red Nu.) sites. **(A)** Control with FB injection into the left side of the spinal cord; **(B)** Group I with FB injection into the left side (unimpaired) of the spinal cord. **(C)** Group II with FB injection into the right side (impaired) of the spinal cord. We analyzed the FB-labeled neurons in both the contralateral (Contra) and the ipsilateral (Ipsi) red nucleus of the control and the rats with neonatal pyramidotomy. **(D,E)** FB-labeled neurons in the red nucleus. Bar = 250 μm **(D)** or 50 μm **(E)**. **(F)** Maps of distribution of the FB-labeled neurons in the red nucleus in the Control, Group I and Group II. Green circles represent the contralateral (to the injection) red nucleus, and the brown circles the ipsilateral red nucleus. **(G)** The mean number of the FB-labeled neurons in the red nucleus. Note that no significant differences were detected in the contralateral red nucleus (green bars) among three groups; however, a significant increase was noted in the ipsilateral red nucleus after neonatal pyramidotomy in comparison to the Control (brown bars). There was no difference in the contralateral or in the ipsilateral red nucleus between Group I and Group II. Plotted white shapes correspond to each rat’s data points. Each rat’s data in the Control group, Group I, and Group II were represented by a particular shape. **p < 0.01, ***p < 0.005.

We also examined rubrospinal connections through retrograde tracing of red nucleus, as shown in Figures 9A–C. The plasticity in the RST was examined by counting the number of FB-labeled neurons (Figures 9D–F) in the red nucleus of the rats with neonatal pyramidotomy and the uninjured controls. There were no differences in the number of retrogradely labeled neurons in the contralateral (to the injection of FB) red nucleus between the rats with neonatal pyramidotomy and the uninjured controls; *F*_(2,17)_ = 2.77, Bonferroni-adjusted *p* = 0.36. On the other hand, the number of retrogradely labeled neurons in the ipsilateral (to the injection of FB) red nucleus was significantly increased after neonatal pyramidotomy; *F*_(2,17)_ = 7.13, Bonferroni-adjusted *p* = 0.02, *f* = 0.92, power = 0.93 (Figure 9G). *Post hoc* testing revealed that Group I (*p* = 0.003) and Group II (*p* = 0.007) rats differed from uninjured controls, but not from each other (*p* = 1.00). The observed changes were in the uninjured hemisphere, and not through the corticoRST in the injured hemisphere, as hypothesized.

### BDA-Labeled Axons in the Cerebral Peduncle and the Dorsal Columns

As explained in the “Materials and Methods” section, the number of BDA-labeled axons in the CST was used to normalize the axon length within the red nucleus and the spinal cord. Similar to our previous studies, there was no significant difference in the number of BDA-labeled axons in the dorsal columns of the control group (68.477 ± 24.757) and Group II (55.951 ± 28.124); *t*_(11)_ = −0.85, *p* = 0.42. Group I has no spinal axons labeled with BDA after neonatal pyramidotomy. In addition, there was no significant difference in the corticospinal axon numbers in the cerebral peduncle between the Control (19.651 ± 7.676) and Group I (23.441 ± 14.846). When BDA was injected into the intact hemisphere (Group II), however, the number of corticospinal axons in the cerebral peduncle (63.615 ± 20.318) was much higher than the Control (19.651 ± 7.676, *p* = 0.001) or Group I (23.441 ± 14.846, *p* = 0.001); *F*_(2,17)_ = 16.70, *p* = 0.0001, *d* = 1.4, power = 1.00. The effect of having a high number of axons in the peduncle on the uninjured side does not affect the testing of the main hypothesis, which examines plasticity in the corticoRST on the injured side. In addition, by correcting axon lengths in the ipsiCST, the effect would be to diminish the raw axon lengths in the ipsiCST.

### Strong Bilateral ICMS Motor Responses in Motor Cortex of the Uninjured Hemisphere

**Figure 10:**
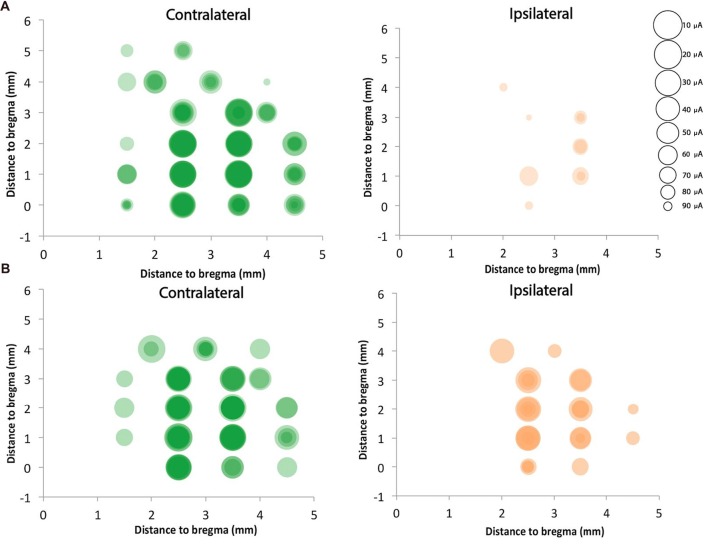
Strong ipsilateral representation after neonatal pyramidotomy. ICMS was performed on four control rats and four rats with neonatal pyramidotomy. Twenty-three sites were mapped in the right cortex, which was in the uninjured hemisphere of rats with neonatal pyramidotomy. Responses from both forelimbs (contralateral and ipsilateral) were recorded. The sizes of the circles were inversely proportional to the threshold for provoking a movement, as indicated in the scale at right. Responses in individual rats were shaded a light color, and the darkness of the circles indicated how many rats of the four rats had responses at each site. In control rats **(A)**, the responses were mainly on the contralateral forelimb. However, in the rats with neonatal pyramidotomy **(B)**, stronger responses were noted in the ipsilateral forelimb in comparison to that of the control rats. In contrast, the responses from the contralateral forelimb of the rats with neonatal pyramidotomy were less robust than that of the control rats.

We mapped motor cortex using ICMS and compared the motor responses from the ipsilateral (to the stimulation) and contralateral (to the stimulation) forepaws of the rats with neonatal pyramidotomy with that of the control rats. In the control rat, we found 14.8 ± 2.9 contralateral responsive sites and only 1.7 ± 1.2 responsive sites from the 23 target sites for each rat (Figures 10A,B). In the injured rat, we detected 11.0 ± 2.4 contralateral responsive sites and 5.4 ± 3.1 ipsilateral responsive sites from the 23 target sites for each rat (Figures 10A,B). The current needed to provoke a movement (threshold) for the ipsilateral paws of the control and injured groups were 47.2 μA and 56.4 μA, respectively. In the case of the contralateral paws of the control and injured groups, the average amount of current stimulating responsive sites were 17.0 μA and 33.5 μA, respectively. We also compared the distribution of the responsive sites between the control rats and the rats with neonatal pyramidotomy using permutation tests. The responses of the ipsilateral paw were more robust in the rats with neonatal pyramidotomy (*p* = 0.04) while the responses of the contralateral paw were more robust in the control rats than those with neonatal pyramidotomy (*p* = 0.03). Thus, the physiology largely mirrors the results of retrograde tracing; very strong connectivity from the uninjured motor cortex to the ipsilateral paws and diminished connectivity to the contralateral paw.

## Discussion

**Figure 11:**
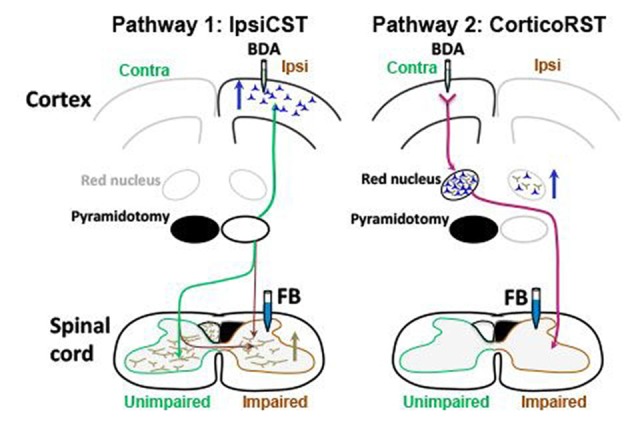
Summary of anatomical results. Blue arrows indicate increased number of neurons retrogradely labeled with FB, and the brown arrow indicates increased axons anterogradely labeled with BDA. The ipsiCST was strengthened in rats with neonatal pyramidotomy. FB-labeled neurons were significantly increased in the ipsilateral (Ipsi) hemisphere when FB was injected into the impaired side of the spinal cord, and BDA-labeled axons were significantly increased in the impaired side of the spinal cord when BDA was injected into the ipsilateral hemisphere. In contrast, there were no differences in the corticoRST circuits from the injured hemisphere. The number of the FB-labeled neurons or the BDA-labeled axons in the red nucleus on the injured side was unchanged. There was an increase of retrogradely labeled neurons in the red nucleus in the uninjured hemisphere from the impaired side of the spinal cord. This is not part of the hypothesized corticoRST recovery circuit.

The anatomical and electrophysiological results favor the ipsiCST over the corticoRST for impaired forelimb control after neonatal injury (Figure 11). In particular, we observed that: (1) the ipsiCST becomes robust after neonatal pyramidotomy and is much stronger than uninjured; (2) the corticoRST after neonatal pyramidotomy does not change significantly; and (3) the cortical representation of the unimpaired forelimb becomes weaker after injury–there may be a cost to bilateral representation. However, the inactivation experiments showed that the rats with neonatal CST injury exhibited deficits after inactivation of motor cortex in both the injured and uninjured hemisphere, while the increased CST connections were observed only from the uninjured hemisphere. These results suggest that the injured hemisphere still participates in control of the motor recovery after neonatal CST injury, even though its descending motor connections do not exhibit plasticity. Thus, there is a striking difference in the anatomical effects of neonatal pyramidotomy, which shows outgrowth only from the uninjured hemisphere, and the behavioral effects of motor cortex inactivation, which demonstrates control from both hemispheres. In this Discussion, we compare the anatomical and physiological results of this study with previous reports of adaptation to motor systems injury and then put forward a hypothesis about why the anatomical and inactivation results differ.

### Anatomical Connections Stronger Only From ipsiCST in the Uninjured Hemisphere

The anatomical results support the ipsiCST as the anatomical pathway with the greatest difference from controls (Figure 11, Pathway 1). CST neurons in motor cortex were significantly increased in the uninjured hemisphere when the retrograde tracer FB was injected into the impaired side of the spinal cord after neonatal pyramidotomy. The anterograde tracing of corticospinal axons corroborated the increased innervation of the gray matter of the impaired side of the spinal cord. In addition, there was a stronger response of the impaired forelimb when ICMS was performed on the uninjured hemisphere. These results all indicate stronger ipsilateral connections from the intact hemisphere to the spinal cord after neonatal pyramidotomy. These connections are a persistence of bilateral projections from each hemisphere that are not pruned in the absence of competition of CST axons from the other hemisphere (Friel and Martin, 2007).

If the uninjured hemisphere is strongly connected to both halves of the spinal cord, how are the two paws represented in the same motor cortex? Our retrograde mapping suggests that the RFA may more strongly encode the ipsilateral forelimb. This is similar to a model of neonatal hemidecortication, in which one cortex is removed. Rats with hemidecortication had significantly increased the number of ipsilateral descending fibers originating from the intact cortex with a higher density of retrogradely labeled neurons in the RFA than the CFA (Umeda et al., 2010). Recently, it has been shown that CFA or RFA control reaching or grasping, respectively (Wang et al., 2017). In adult rats with unilateral CST injury, we have demonstrated that electrical stimulation of motor cortex in the uninjured hemisphere promotes recovery of skilled locomotion through ipsilateral control (Carmel et al., 2014). Given the disproportionate representation of the impaired forelimb in the RFA, this site could be specifically targeted to improve motor control after neonatal injury.

In contrast to the large-scale adaptations in the ipsiCST, there was little evidence for adaptation in the corticoRST from the injured hemisphere. We did not observe plasticity in corticorubral projections found by others (Z’Graggen et al., 2000). Not only was there no increase in corticorubral connections, the axon length in the red nucleus in the injured hemisphere was about a third of that in control rats. This strong trend does not support this connection as an important site of anatomical plasticity. There was some plasticity in the RST, with more retrogradely labeled neurons in the red nucleus on the same side as injection of FB into the spinal cord (Figure 9G), but no plasticity in the main crossed RST. Thus, the anatomical results strongly support the uninjured hemisphere, but not the injured hemisphere, as the origin of descending motor connections after neonatal pyramidotomy.

If descending motor connections originate primarily from the uninjured hemisphere in rats with neonatal pyramidotomy, then why did inactivation of cortex in both hemispheres cause worse performance on the knob supination task? Our hypothesis is that muscimol infusion inactivates both motor and sensory circuits. Unlike the motor systems, the sensory systems that are critical for movement (those mediated by the dorsal column-medial lemniscus system) do not project bilaterally early in development and, therefore, do not show large-scale adaptation to unilateral injury. Thus, rats with neonatal pyramidotomy likely have motor cortex connectivity from the uninjured hemisphere and sensory cortex connectivity to the injured hemisphere. The sensory and motor cortices overlap in all mammals, but this overlap is particularly strong in the rat (Barthas and Kwan, 2017). Muscimol inactivation in the center of the CFA likely impairs movement because of both sensory and motor impairments. In children with congenital unilateral cerebral palsy, we have recently compared the anatomy and physiology of the CST and dorsal column-medial lemniscus system. We observed that disruptions of sensory connectivity more strongly predicted hand movement impairments than motor connectivity (Gupta et al., 2017). This possibility can be tested by measuring sensory function before and after inactivation in these rats. In addition, pathway-specific inactivation of motor and sensory systems (Park and Carmel, 2016) can help to discriminate these possibilities.

We used seven rats with neonatal pyramidotomy for inactivation of motor cortex; however, only three rats had complete lesions, and four rats had incomplete lesions (20%, 14%, 33%, 30% sparing, Figures 2C4–7). The question is whether partial sparing affects the results of motor cortex inactivation. To answer this, we compared the effects of inactivation only in the three rats with complete lesions (Figure 4, circles; see “Results” section for statistics). These rats had significant and large-scale impairments in the right forepaw with right motor cortex inactivation, an effect that was not observed in uninjured rats. Rats with partial injury (Figure 4, triangles) had similar magnitude of deficits with inactivation of each hemisphere to rats with complete lesions. This is similar to previous results showing motor impairments in rats with >70% interruption of the CST (Whishaw et al., 1993). This suggests that motor system adaptation is similar in rats with complete vs. >67% interruption of the CST. We have not tested this hypothesis in this study, since only rats with complete pyramidotomy were used for the anatomical studies. This will be crucial to understand in studies that mimic clinical brain injuries, which often spare some CST connections (Kuo et al., 2017).

It is also reported that the spared supplementary motor cortex residing in the lesioned hemisphere controlled the spontaneous recovery following lateral frontal motor injury in monkey (McNeal et al., 2010) and transient focal ischemia in the rat (Mitchell et al., 2016). Moreover, it has been addressed that the ipsilateral projections might increase corticospinal excitability thereby supporting the recovery process of the primary pathways in patients with an at least partially spared CST (Stoeckel and Binkofski, 2010). Furthermore, it has been shown that there was persistent bilateral activation and increased contralesional connectivity in patients with subcortical lesions and good outcomes (Gerloff et al., 2006). These results suggest that the injured hemisphere or the projections from the injured hemisphere also made a contribution to motor recovery after CST injury.

Besides the ipsiCST and the corticoRST, other spared subcortical motor pathways such as cortico-reticulospinal tract, can also mediate motor control after CST lesion. There were approximately three times as many axons in the cerebral peduncle when BDA was injected into the uninjured hemisphere of rats with neonatal pyramidotomy compared to the injured hemisphere or the control rats. In addition to the red nucleus on the uninjured side, another potential target for the axons in the peduncle may be reticulospinal neurons. CST lesions caused plasticity and remodeling of the reticulospinal tract (Zörner et al., 2014; Baker et al., 2015; García-Alías et al., 2015; Baker and Perez, 2017), suggesting a possible role in recovery from neonatal injury.

In the current study, we used several behavioral tests including pasta handling, horizontal ladder walking, and supination task to test movement, but we did not examine sensory function after neonatal pyramidotomy. It has been reported that the sensory function may be impaired after pyramidotomy in adult mice (Starkey et al., 2005) and rats (Thallmair et al., 1998) by using the tape removal test, which was originally developed to assess somatosensory asymmetry and sensory function after sensorimotor cortex lesions. However, it is unclear if such sensory function impairment also occurs after neonatal pyramidotomy; this is another limitation of our study.

The strong ipsilateral connections from the uninjured hemisphere may be accompanied fewer contralateral connections. There was an unexpected decrease in the number of retrogradely labeled neurons for the crossed CST from the rats with neonatal pyramidotomy compared with controls (Figure 6I). The motor map of the contralateral forelimb was also less robust in the rats with neonatal pyramidotomy (Figure 10). Curiously, the amount of spinal axon labeling for the contralateral CST was not different between the rats with neonatal pyramidotomy and uninjured controls (Figures 7E,F). The difference in the retrograde and anterograde results could be due to the contralaterally projecting neurons extending longer gray matter terminations in the spinal cord. However, the behavioral tests, especially ladder walking, did not show deficits of the contralateral forelimb after neonatal pyramidotomy. This finding suggests that the reduction of the contralateral connections was not enough to cause behavioral deficits in movement of the contralateral forelimb. Whether these changes may have subtle functional consequences is an important question to answer in order to determine the limits of motor system adaptation.

Finally, the pyramidotomy has advantages for testing circuit adaptation to injury, but it does not mimic the pathology of neonatal brain injury. The advantage of this model is that it specifically affects one tract in comparison to most other preclinical models of spinal injuries often affecting multiple tracts. Another advantage is the reproducibility of the CST lesion and its subsequent behavioral deficits (Lee and Lee, 2013; Kathe et al., 2014; Fink and Cafferty, 2016). Perhaps most importantly, pyramidotomy tests the limits of adaptation in the two circuits we studied by completely removing one half of the system and by preserving all of the motor cortex to red nucleus connections on the side of injury. However, the lesion does not mimic the lesions we observe clinically in children with hemiplegia, especially periventricular and middle cerebral artery territory infarcts (Gupta et al., 2017). An important next goal is to understand the alterations in these circuits in models of these clinical lesions. These studies would set the table for trials to strengthen spared descending motor pathways to restore function after unilateral injury to the developing CST.

## Author Contributions

T-CW: design of the experiment, conducting the experiment, data collection, drafting the article. SL: conducting the experiment, data collection and analysis, drafting the article. CP: conducting the experiment, data collection and analysis. JM and NJH: data analysis. DG: data analysis, drafting the article. SR-G: data collection and analysis, drafting the article. JB, LG and MK: data collection. JBC: design of the experiment, data collection and analysis, drafting the article.

## Conflict of Interest Statement

The authors declare that the research was conducted in the absence of any commercial or financial relationships that could be construed as a potential conflict of interest.
