# Corrigendum: Plasticity in One Hemisphere, Control From Two: Adaptation in Descending Motor Pathways After Unilateral Corticospinal Injury in Neonatal Rats

**DOI:** 10.3389/fncir.2018.00080

**Published:** 2018-09-25

**Authors:** Tong-Chun Wen, Sophia Lall, Corey Pagnotta, James Markward, Disha Gupta, Shivakeshavan Ratnadurai-Giridharan, Jacqueline Bucci, Lucy Greenwald, Madelyn Klugman, N. Jeremy Hill, Jason B. Carmel

**Affiliations:** ^1^Motor Recovery Laboratory, Burke-Cornell Medical Research Institute, White Plains, NY, United States; ^2^Departments of Neurology and Pediatrics, Brain and Mind Research Institute, Weill Cornell Medicine, Cornell University, New York, NY, United States

**Keywords:** plasticity, corticospinal tract, rubrospinal tract, deficits, neonatal pyramidotomy, rats

An author name was incorrectly spelled as Madelyne Klugman. The correct spelling is Madelyn Klugman. The authors apologize for this error and state that this does not change the scientific conclusions of the article in any way.

The original article has been updated.

## Conflict of interest statement

The authors declare that the research was conducted in the absence of any commercial or financial relationships that could be construed as a potential conflict of interest.

